# Leaders Are More Attractive: Birds with Bigger Yellow Breast Patches Are Followed by More Group-Mates in Foraging Groups

**DOI:** 10.1371/journal.pone.0026605

**Published:** 2011-10-19

**Authors:** Zoltán Tóth, Matteo Griggio

**Affiliations:** Konrad Lorenz Institute for Ethology, Department of Integrative Biology and Evolution, University of Veterinary Medicine of Vienna, Vienna, Austria; University of Bristol, United Kingdom

## Abstract

Social network theory provides a perfect tool to better understand the population-level consequences of how individuals interact and make their decisions; however, this approach is generally overlooked among evolutionary biologists interested in social relationships. Here, we used social network analysis to examine the patterns of leader-follower interactions in relation to individual characteristics in foraging groups of free-living rock sparrows (*Petronia petronia*). We found that yellow feather ornamentation, a carotenoid-based trait, was the best predictor of leadership: birds with bigger ornaments exerted greater influence in the foraging groups and were followed by more group-mates than less elaborate individuals. An individual's tendency for eliciting followings was not influenced by sex, condition or the level of parental investment. None of the above individual characteristics had significant effect on the tendency of individuals to follow others. Our results indicate that a sexually selected trait can also play a significant role in group coordination and social organization of a species.

## Introduction

The investigation of leadership and decision-making in animal groups is of great interest from both evolutionary and behavioural perspectives, as understanding of the process of how leaders and followers emerge in animal societies can greatly improve our knowledge about how social units evolve, function and persist in different contexts (reviewed in [1]–[3]). In the last decade, numerous theoretical studies have investigated the circumstances in which decision-making is expected to be based on consensus or despotism [4], [5], the way in which the simple rule of thumbs that individuals follow in a group may lead to complex dynamics [6], and whether special traits of individuals may increase their propensity to act as a leader in coordination problems [7], [8]. Empirical studies have provided further information about contexts and species in which the predictions of particular theories hold or fail (e.g. [9], [10]).

Foraging in groups is known to be advantageous in terms of increased likelihood of finding food, detection and avoidance of predators [11], but it is less obvious how coordination between group-mates is achieved especially if foraging groups are heterogeneous as a consequence of individual variation in behaviour, physiology or social status [12], [2]. For instance, in three-spined sticklebacks, *Gasterosteus aculeatus,* individuals preferred to associate with bold group-mates [13], whereas in zebra finches, *Taenopygia guttata*, more active and explorative birds were more likely to act as leaders in foraging pairs [9], [14]. Other studies have supported the hypothesis that differences in energy reserves or need of particular resources can lead to the emergence of different behavioural roles in foraging groups, and individuals with lower resources tended to initiate and/or coordinate foraging (e.g. Atlantic salmon, *Salmo salar*: [15]; plains zebra, *Equus burchellii*: [16]). In other cases, dominant individuals (e.g. gray wolf, *Canis lupus*: [17]; chacma baboons, *Papio ursinus*: [18]) or on the contrary, subordinates (e.g. black-capped chickadee, *Parus atricapillus*: [19]), or individuals with more detailed information about food resources (e.g. golden shiner, *Notemigonus crysoleucas*: [20]) were found to elicit followings and lead foraging bouts more often. Even if some of these traits are interrelated, e.g. subordinates may have generally lower reserves [8], or more explorative individuals may have more detailed information about their environment [21], these findings indicate that leader-follower relationships in foraging groups are of complex origin in the animal kingdom and leadership cannot be explained by a single physiological or behavioural characteristic. More likely, there are various possible mechanisms that lead to the emergence of leaders under different environmental, cognitive and social constraints [10].

Social network analysis is the study of social groups modelled by networks of individuals connected by social relationships [22]. It is an increasingly popular tool for the study of animal social structure, because it provides a robust methodology for studying the complexity of social behaviour from individual to population level (for detailed reviews, see [23]–[26]). Using network derived quantitative metrics, several studies have investigated how topological positions are related to different individual traits, mostly in fish and mammals (e.g. [10], [27]–[29]), and only a few bird species [30], [31]. Some of these studies have also demonstrated that the application of the network approach offers new insight how leaders acquire their status through social relations in the network of interacting individuals (e.g. [32]). Thus, social network analysis can be a perfect tool to examine the ultimate consequences of how individuals interact and make decisions during different social activities.

The rock sparrow, *Petronia petronia* (Linnaeus 1776), is a small, cavity-nesting passerine species resident in Southern Europe, North Africa, and South West Asia; they usually breed at low densities in small and loose colonies near human settlements [33]. A yellow breast patch, a carotenoid-based trait present in both sexes, plays an important role in the reproductive period signalling attractiveness and social status in the species [34], [35]. Field observations and experiments in captivity demonstrated that both males and females prefer mates with a large yellow patch over individuals with a small yellow patch [34]–[37]. The dimensions of the patch are also important in both male-male and female-female aggressive interactions as an indicator of the competitor's quality [35], [38]. Therefore, the same visual signal is likely to have a dual utility of armament (badge of status) and ornament in both sexes [38]. Rock sparrows are also characterized by several forms of parental care: both biparental and uniparental care (both sexes may desert the brood during the nestling feeding stage) occur in the species. In the latter case, one parent is able to provide sufficient provision for the offspring on its own [39]–[41]. Despite the intensive research on rock sparrows' reproductive behaviour, however, we know little about the social organization of their breeding colonies and foraging groups.

In this study we used social network analysis to investigate leader-follower interactions in foraging groups of rock sparrows during the reproductive period. More specifically, we tested how individual characteristics affect birds' tendency to elicit followings from their group-mates. We hypothesized that either i) body condition, ii) frequency of offspring provisioning, or iii) feather ornamentation can be influential and may increase the probability of acting as a leader and initiate foraging bouts. According to theory [7] birds with lower body condition may emerge as leaders simply because of their more frequent need of foraging; in that case individuals with lower reserves show higher feeding activity and thus are predicted to elicit followings more often. In a similar way, better provisioning parents can also be expected to induce more followings; in this case their higher feeding activity originates from their increased parental investment/effort. On the other hand, feather ornamentation can also be an important predictor of leadership if, by following good quality individuals (in terms of social status or attractiveness), birds may gain future mating [31] or present foraging benefits [42]. Such foraging benefits can be expected if more ornamented individuals have better foraging abilities (in terms of locating resources or solving a foraging problem); such a relationship has been demonstrated in another passerine species with carotenoid-based colouration [42]. We hypothesized that differences in the above individual characteristics (i.e. body condition, frequency of offspring provisioning and feather ornamentation) can create natural heterogeneity among breeding rock sparrows and may be associated with the pattern of followings in their foraging groups.

## Methods

### Ethics Statement

All handling and ringing was performed by expert ringers provided with the appropriate ringing permits issued by the Istituto Nazionale Fauna Selvatica (now ISPRA, Ozzano Emilia, Italy). The long-term nature of the study allowed us to confirm that handled birds and their offspring did not suffer any detectable reduction in welfare and survival.

### Study subjects

We studied a northwest Italian alpine population of Rock Sparrows in the higher Susa valley, as part of a wider long-term study of rock sparrow ecology [39], [43]–[45]. From 1991, an average of 40 nestboxes (range 36–51) was set up in three neighbouring villages (44°56′ N, 6°48′ E; the two furthest villages were ca. 2 km) surrounded by pastures and mountain meadows. The nest-boxes were installed approximately 30 m from each other in each village and checked every 2–3 days during the entire breeding season to identify pair bonds and determine the breeding success. The breeding population consisted of approximately 20 pairs in each year. Usually, parents were trapped at the nest when feeding the young, but a few individuals were also trapped at the nest before the onset of reproduction. For each individual within a breeding season, badge size (as the length of the major axis of their yellow patch; for details see [40]), weight and tarsus length was measured. Adults were individually colour ringed. More details about the population studied are given in [40], [41].

### Behavioural observations

In 1999, 2000 and 2002 (“study years” henceforward), we observed the foraging behaviour of adults for 1 h every 2–3 days, in the morning and late afternoon (060-1030 and 1530–1900 hours, respectively), throughout the breeding season (late May till late July) with 20–60× spotting scopes while sitting in the open, approximately 30–50 m from the nests. During these observations, we recorded i) the number of nestling feeding trips of the parents (in total we observed 43 pairs; for more details see [46]), and also ii) the identity of individuals in the foraging groups (Table 1). Foraging typically took place at mowed patches of the meadows in the area where nestboxes were installed (typically 3–4 patches in each study year) or in other places outside the nestboxes area (several additional patches); on these spots, insects were likely to be more accessible for rock sparrows. These distinct patches were between ∼0.05 and ∼5 km apart from each other. Within the groups of foraging birds, we defined ‘following’ as an event when an individual started a foraging bout (arrived at a feeding place either from a nestbox, resting place or another patch) and was followed by one or more group-mates. The former individual was described as ‘initiator’, while the latter(s) as ‘follower(s)’. Only those following events were taken into account in which the follower bird both followed the initiator within 5 s and arrived within 0.5 m of it.

**Table 1:** Characteristics of the observed foraging groups.

	1999	2000	2002
Sampling period	14 June – 25 July	5 June – 28 July	20 June – 28 July
No of sampling days	10	8	12
No of observed individuals (males/females)	19 (11/8)	20 (12/8)	16 (8/8)
No of observed foraging groups	10	14	13
Average group size	3.40±0.52	3.42±0.51	3.46±0.52
Identification efficiency (both participants of the followings within groups)	57.14%	65.38%	53.45%
No of identified followings	24	34	31

### Network construction

**Figure 1:**
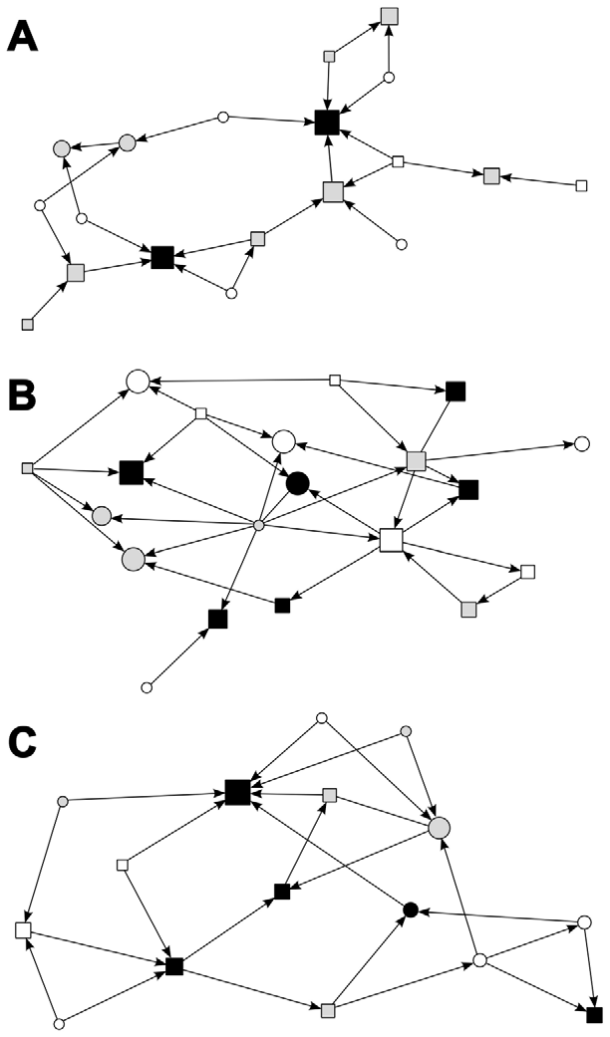
Constructed following networks in 1999 (a), 2000 (b) and 2002 (c). Individuals are represented by nodes with sex indicated by nodes’ shape: males are squares and females are circles. The colour of the nodes reflects individuals’ badge size: small (white; 8.70–12.23 mm), middle-sized (grey; 12.24–15.76 mm) or large (black; 15.77–19.30 mm). Please note that we only created these badge size categories for demonstration; we used badge size in our analyses as a covariate. Node size is proportional to in-degree (i.e. the number of followers). Directed links from one node to another indicate the occurrence of following between the two individuals. Graphs were generated using spring-embedding algorithm with 10000 iterations in NetDraw [71].

We constructed social networks based on followings within the foraging groups in each study year (Fig. 1). Only those followings were considered in which both participants were unambiguously identified. Followings within reproductive pairs were excluded, otherwise sexual interactions such as mate guarding could have influenced our analysis. In the constructed networks individuals are represented by nodes that are connected to each other by ties (*i.e*. following interactions). As followings were rare events (mean number of followings per dyad: 0.09±0.31, range: 0–2, across all years), a tie was considered to exist between two birds if one followed the other one at least once during the sampling period in a study year (Table 1), and the direction of the tie was taken to be from the ‘follower’ toward the ‘initiator’ individual. By doing so, we built binary directed networks (binary because a tie is either present or absent between a pair of nodes, and directed in that a tie directing from A toward B does is not identical to a tie directing from B to A).

We used several network properties to characterize the observed following networks: overall reciprocity, overall transitivity (*T*), the mean size of influence domain (*I*_i_) and the variance of in-degree (*k*_in_) and out-degree (*k*_out_; using the relevant functions of ‘*igraph*’; [47] and ‘*tnet*’; [48], packages, run with *R* 2.13.0 Statistical Program [49]. Overall reciprocity was calculated as the proportion of pairs of connected nodes between which there are ties in both directions; its value indicates the extent to which followings were mutual between group-mates. Transitivity is the proportion of transitive triples (if A directs a tie to B and B directs a tie to C, then A also directs a tie to C) in a network, reflecting the tendency of nodes to form clusters or tightly connected groups within the network [50]. The influence domain of a node is the set of nodes that are directly or indirectly linked to a given node; its mean size reflects the average number of nodes that can reach another node in the network. In-degree is the number of direct ties a node receives from its neighbours (i.e. number of followers), whereas out-degree is the number of ties a node directly sends to other nodes in the network (i.e. number of followed group-mates). To test whether the characteristics of the observed networks could emerge by chance, i.e. they are simply the outcome of random followings within foraging groups, or not, we compared them to those of equivalent random networks. Random networks contained the same number of ties and nodes, and were generated by randomly reassigning connections among pairs of nodes [51], [52]. To compare the values of metrics in the observed and equivalent random networks, we performed two-tailed Monte Carlo tests with 10,000 resamplings. We defined an observed value to be significantly different from random if it fell within the top or bottom 2.5% of the distribution for the statistic obtained from the generated random networks [53].

### Statistical analyses

To investigate the relationship between birds’ position in the network and individual characteristics, we computed three node-based metrics that express different aspects of topological importance. In these analyses we used a fully independent, reduced data set by combining the data of those individuals that were observed only in one study year (37 out of 45 birds). Degree prestige (*P*_D_) was defined as the in-degree of each node divided by *n*-1, where *n* is the number of nodes in the network (thus degree prestige is identical to normalized in-degree; [54]); it yields the proportion of nodes in the network from which given node directly receives ties. Proximity prestige (*P*_P_) of a node was computed by dividing given node’s influence domain (expressed as a proportion) by the average distance from all nodes in the influence domain [54]. This prestige score increases as influence domain becomes larger and/or average distance gets smaller. Proximity prestige is maximal if a node is directly reachable by all other nodes in the network, whereas nodes without influence domain get zero (minimum) proximity prestige by definition. We also calculated degree centrality (*C*_D_) as the out-degree of each node divided by *n*-1 (thus it is equivalent to normalized out-degree), reflecting the proportion of nodes to which given node directly sends ties [54]. As measures derived from relational data violate the assumption of independence in parametric statistical analyses [24], we used randomizations in all the applied tests with 5000 iterations to calculate simulated probabilities. First, we applied permuted Pearson correlation tests (‘*simba*’ R package [55]) to investigate how the above metrics correlate with each other in the three networks. Second, we applied linear models with Monte Carlo simulations (‘*pgirmess*’ R package [56]) to assess the effect of three individual characteristics on the calculated metrics: body condition (calculated according to [57]), number of feeding trips per hour (as a proxy for parental investment; calculated as described in [41]), and badge size (measured as in [34], [35]). We included the possible explanatory variables as covariates into the candidate models together with ‘sex’ (as a factor) and its interactions with each explanatory variable. Also, we included ‘year’ (as a factor) into the models to account for any variation between the studied years. We compared all possible combinations of predictors using the information-theoretic approach [58] based on AIC_c_ (Akaike’s Information Criterion corrected for small sample size; Table 2). We evaluated each candidate model by its Akaike weight (ω; reflecting the probability that a given model is actually the best in the model set). For the predictors in the best model, we estimated effect sizes (see [59], [60]) as the proportion of variance explained by each trait, *i.e*. *η* ^2^ and its 95% confidence interval [61]. For the correlation tests in the three networks we applied sequential Bonferroni-correction for significance levels, in all other tests α was set to 0.05. Tests were two-tailed throughout the analyses.

**Table 2:** Network properties for real (observed) and random networks constructed from followings in foraging groups in the 3 years.

		1999	2000	2002
Overall reciprocity	Observed	0	0	0
	Random	0.03±0.04	0.04±0.04	0.05±0.05
	*P*	0.931	0.481	0.494
Overall transitivity	Observed	0.22	0.16	0.08
	Random	0.07±0.055	0.08±0.04	0.10±0.05
	*P*	0.029	0.086	0.815
Mean size of influence domain	Observed	1.63	3.40	5.44
	Random	4.54±1.60	8.94±2.59	7.16±2.09
	*P*	<0.001	0.004	0.472
Variance of in-degree	Observed	2.40	1.41	2.80
	Random	1.14±0.37	1.47±0.46	1.41±0.48
	*P*	0.011	1	0.025
Variance of out-degree	Observed	0.84	4.78	0.66
	Random	1.23±0.39	1.47±0.46	1.40±0.49
	*P*	0.392	<0.001	0.107

Values for the random networks are means (with SD) of 10000 iterations. Two-tailed *P* values were obtained by Monte Carlo randomization tests.

## Results

### Characterization of the observed following networks

Nodes were sparsely connected as indicated by low tie density (number of existing ties divided by the number of all possible ties) in all observed networks (0.07, 0.08 and 0.10, respectively). There were no reciprocal ties present in either study year, indicating that followings were typically unidirectional, although this characteristic was not significantly different from what can be expected under randomly occurring followings (Table 2). We found significantly higher transitivity in 1999, but not in other years, compared to that of random networks. That indicates more tightly clustered groups of individuals only in one specific year. Both in 1999 and 2000, the mean size of influence domain was lower than in random networks, suggesting that individuals were less reachable by others in these years, but we found no such difference in 2002. In 1999 and 2002 the variance of in-degree (the number of followers), while in 2000 the variance of out-degree (the number of followed group-mates) was significantly higher than in the random networks. These findings indicate that the social structure of the foraging groups of rock sparrows is likely to vary between years, shows no consistent difference from random from several aspects and shows no distinct structural characteristics (*e.g.* no evidence for the presence of tightly connected subgroups of individuals). On the other hand, degree distributions were found to be non-random from at least one perspective in all study years and the significantly higher variation of in-degree in two study years also suggests that significant differences in leadership (*i.e*. tendency to elicit following among group-mates) can emerge between individuals in the following networks of foraging birds.

### Nodal metrics of topological importance and their relationship with individual characteristics

In all three networks degree prestige and proximity prestige were positively correlated with each other, indicating that individuals who had more followers in the groups were also more proximate to group-mates within their influence domain (Table 3). On the other hand, degree prestige indices were negatively correlated to degree centrality, *i.e.* the more prestigious individuals were, the less frequently they followed other group-mates in all study years. Proximity prestige was also significantly negatively correlated to degree centrality in two study years, but this correlation was marginally non-significant in 2002 after sequential Bonferroni-correction (with α = 0.017).

**Table 3:** Permuted correlation tests between degree prestige (*P*_D_), proximity prestige (*P*_P_) and degree centrality (*C*_D_) in the 3 networks.

	1999			2000			2002		
	*N*	*r*	*P*	*N*	*r*	*P*	*N*	*r*	*P*
*P*_D_ vs. *P*_P_	11	0.99	<0.001	12	0.93	<0.001	14	0.85	<0.001
*P*_D_ vs. *C*_D_	11	−0.72	0.004	12	−0.76	<0.001	14	−0.68	0.010
*P*_P_ vs. *C*_D_	11	−0.74	0.007	12	−0.83	<0.001	14	0.57	0.019

*N* is sample size (i.e. the number of individuals in the networks), *r* is Pearson’s correlation coefficient.

**Figure 2:**
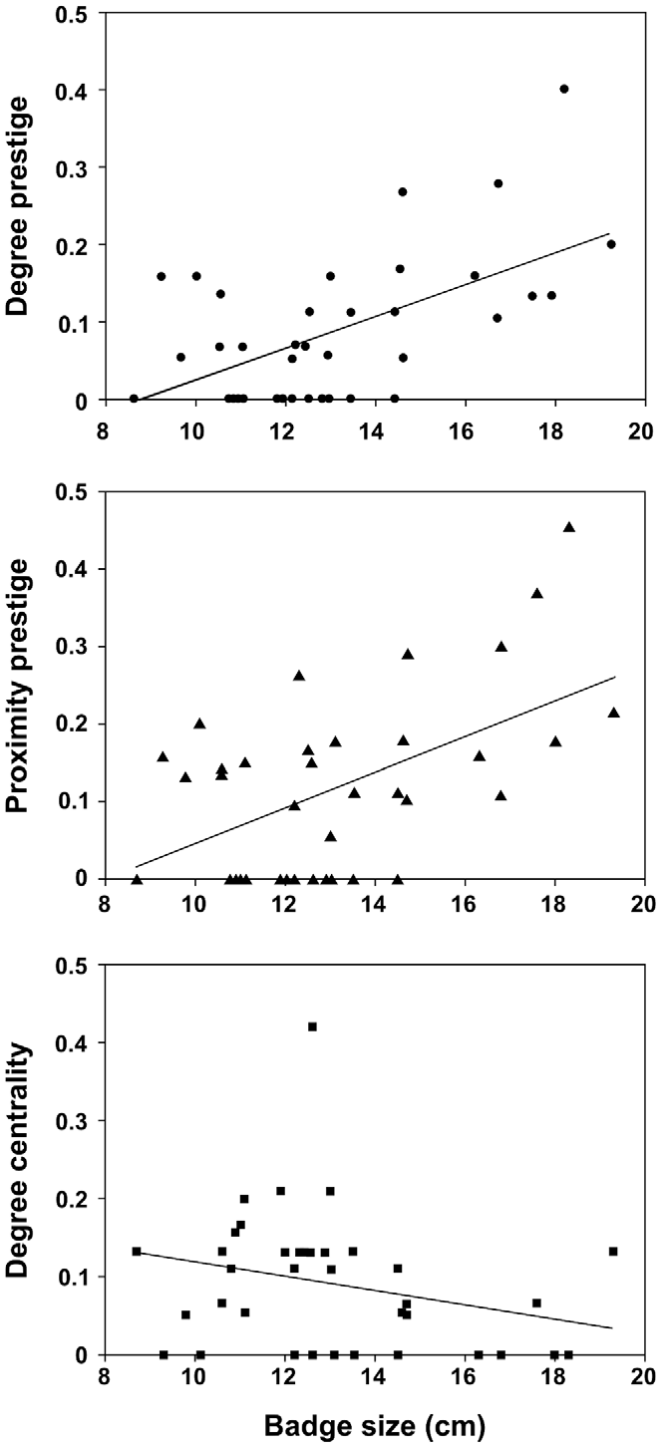
Relationship between badge size and the calculated three nodal metrics. Degree prestige (a) and proximity prestige (b) are indices reflecting how important is an individual in terms of initiating followings in the foraging groups, whereas degree centrality (c) indicates the tendency of an individual to follow others.

The most important predictor of prestige was badge size (Table 4, Fig. 2): individuals with larger badges were followed proportionally by more group-mates (degree prestige *versus* badge size: *F*_1,35_  = 16.97, *ŋ* ^2^ [CI]  =  0.33 [0.09–0.52], *P*<0.001) and also, the average distance between them and their followers was shorter (proximity prestige vs. badge size: *F*_1,34_  =  13.03, *ŋ* ^2^ [CI]  =  0.28 [0.06–0.48], *P*  =  0.001). Proximity prestige was also affected by year (proximity prestige *versus* year: *F*_1,34_  =  6.05, *ŋ* ^2^ [CI]  =  0.15 [0.003–0.36], *P*  =  0.021), indicating the presence of annual variation in average distance between leaders and followers. Badge size was also the best predictor of degree centrality, *i.e.* individuals’ tendency to follow others, but its effect was not significant (*F*_1,35_  =  2.83, *ŋ* ^2^ [CI]  =  0.07 [0–0.27], *P* = 0.105).

**Table 4:** The 5 best-fitting linear models for each dependent variable.

Dependent variable	Model	AIC_c_	δAIC_c_	ω
Degree prestige (*P*_D_)	Badge size	−78.788	0	0.366
	Year + Badge size	−76.786	2.002	0.135
	Sex + Badge size	−76.416	2.372	0.112
	Year + Body condition + Badge size	−74.4	4.389	0.041
	Sex + Body condition + Badge size	−74.28	4.509	0.038
Proximity prestige (*P*_P_)	Year + Badge size	−63.457	0	0.311
	Badge size	−61.946	1.511	0.146
	Year + Trips/h + Badge size	−61.209	2.248	0.101
	Year + Body condition + Badge size	−60.908	2.549	0.087
	Year + Sex + Badge size	−60.805	2.651	0.082
Degree centrality (*C*_D_)	Badge size	−71.588	0	0.141
	- (*no predictors included*)	−71.085	0.503	0.109
	Year + Badge size	−70.826	0.762	0.096
	Year + Body condition + Badge size	−70.166	1.423	0.069
	Year	−69.806	1.783	0.058

AIC_c_ is Akaike’s information criterion corrected for small sample size, δAIC_c_ is the difference in AIC_c_ values between the best model and a given model, ω is Akaike weight reflecting the probability that a given model is the best in the model set.

## Discussion

In this study we investigated the effect of several individual characteristics on behavioural roles (leader *versus* follower) that individuals may play during social foraging in free-living rock sparrows. We found that badge size was the best predictor of leadership in foraging groups: individuals with larger yellow badges were followed by more group-members, and therefore exerted greater influence on initiating foraging bouts. An individual’s tendency to elicit followings was not affected by sex, body condition or the level of parental investment; similarly, none of the above individual characteristics influenced the propensity of birds to follow others. Our results indicate that social status and attractiveness is of great importance not only in sexual selection and mating context, but it also affects social foraging decisions in this species.

Previous findings on the significance of badge size in rock sparrows showed that individuals with larger breast patches are more successful during aggressive interactions and they are the preferred mating partners in both sexes [35], [37]. The importance of ornaments in signalling individual quality has long been recognized [62], but their effect on social organization has been rarely investigated. A study on house finches [31] showed that less attractive males were more socially labile and associated more frequently with members of distinct social groups than more attractive males. By doing so, less attractive males actively sought social environments where their mating opportunities could be more favourable. However, in our study the observed pattern in followings cannot be explained by similar mating benefits. Although preference for larger badge size can be a possible confounding factor, following more attractive individuals would entail with any reproductive benefits if followings occurred mostly between different sexes. However, ∼ 54% (ranging from 65% to 48% in the three years) of the followings occurred in same-sex (both male-male and female-female) dyads; besides, we found no significant relationship between badge size and tendency to follow others either (to the contrary of what we would expect according to [31]).

Instead of possible mating benefits, the observed pattern in leadership may be related to other advantages: following good quality individuals can be beneficial during social foraging if it increases the probability to find novel or good quality, such as carotenoid-rich food. It is known that the breast patch of rock sparrows is carotenoid-based and condition dependent [63]. As these pigments can be only obtained from the diet [64], parents may increase the amount of carotenoid-rich food they can deliver to their nest by preferably following more attractive individuals. Carotenoids are important antioxidants and are especially crucial in periods of intense growth (such as embryo development or nestling phase) for prevention of oxidative stress and regulation of immune function [65]–[67]. For example, Biard *et al.* (2006) [68] found that experimentally-manipulated availability of carotenoids in the diet had important fitness consequences in blue tit (*Parus caeruleus*) and great tit (*Parus major*) nestlings. Thus, breeding individuals may benefit from following more elaborated group-mates if, by doing so, they can improve the quality of their offspring’s or alternatively their own diet. In the siskin, *Carduelis spinus* (a passerine species which is also characterized by carotenoid-based colouration), it has been shown that individuals with longer yellow wing stripes may solve a foraging problem faster thus shows increased foraging abilities than less elaborated individuals [42]. If such relationship between feather ornamentation and foraging ability also exists in rock sparrows, following more elaborated group-mates may be beneficial in terms of foraging efficiency and/or success. However, as the proposed relationship has not been investigated explicitly in our study, future studies are required to test the validity of the above hypothesis in this species under experimental conditions. Alternatively, rock sparrows may follow individuals with bigger breast patches more often because of their social status, although we have no clear explanation why following dominant individuals would be beneficial in a foraging context.

We did not find any effect of either body condition or nestlings’ feeding rate on leadership role, despite the strong theoretical background [7], [69]; both characteristics were assumed to be influential on the basis of more frequent foraging trips (because of own needs in the former case, whereas due to the nestlings’ needs in the latter). It is possible that differences in these individual traits were less detectable for conspecifics during the breeding period, or alternatively, feather colouration was a stronger (visual and/or social) stimulus for foraging individuals. There are several examples for socially mediated leader-follower interactions [13], but according to our knowledge, this is the one of the few studies that found direct relationship between ornament elaboration and leadership in foraging groups of a vertebrate species. Our results are in accordance with another study on siskins [70], where more brightly ornamented birds showed increased leadership capabilities, i.e. gave fewer contact calls when isolated and were attracted to the feeding area less often by decoys than less ornamented individuals. From an evolutionary point of view, these finding implies that a sexually selected trait can be important not only in intrasexual competition and mating success, but in group coordination and social organization as well. According to this assumption, carotenoid-based feather colouration may signal several aspects of good quality in rock sparrows, and preference for more elaborated individuals seems to affect the social dynamics of their foraging groups as well.

In conclusion, we used field data collected in three years to investigate leader-follower interactions in foraging groups of free-living, breeding rock sparrows. Our study revealed that preference for attractive group-mates is present not only in reproductive, but also in a social foraging context. Despite the low sample size, we found a clear relationship between breast patch size and leadership: individuals with bigger badge patches elicited followings more often than less-ornamented individuals in the foraging groups. As any mating benefit originating from following more attractive group-mates was unlikely (due to the high proportion of same-sex interactions), we assume that the observed pattern may be related to certain foraging benefits, such as increased efficiency or success.
